# Effect of Boron-Doped Mesoporous Bioactive Glass Nanoparticles on C2C12 Cell Viability and Differentiation: Potential for Muscle Tissue Application

**DOI:** 10.1021/acsbiomaterials.2c00876

**Published:** 2022-11-15

**Authors:** Duygu Ege, Qaisar Nawaz, Ana M. Beltrán, Aldo R. Boccaccini

**Affiliations:** † Institute of Biomedical Engineering, Boğaziçi University, Rasathane Street, Kandilli, İstanbul34684, Turkey; ‡ Department of Materials Science and Engineering, Institute of Biomaterials, University of Erlangen-Nuremberg, 91058Erlangen, Germany; § Departamento de Ingeniería y Ciencia de los Materiales y del Transporte, Escuela Politécnica Superior, Universidad de Sevilla, 41011Seville, Spain

**Keywords:** MBG, bioactive glass, myotube, myoblast cell, fluorescence microscopy, actin

## Abstract

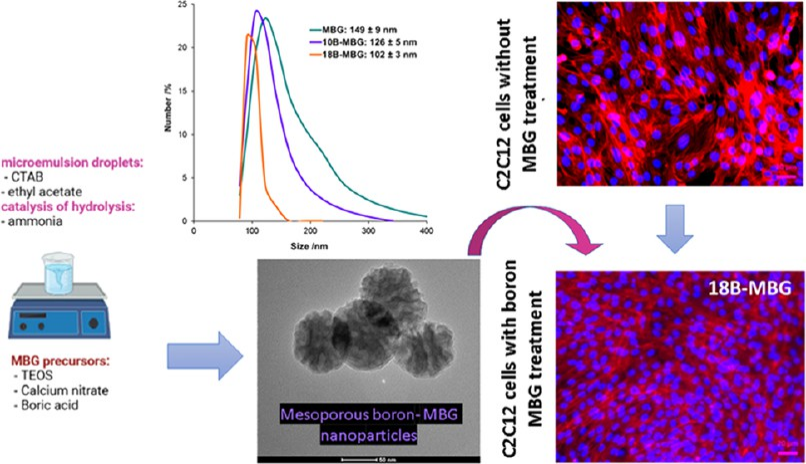

Mesoporous bioactive glasses (MBGs) exhibit a high surface area and a highly ordered mesoporous structure. MBGs have potential for both hard and soft tissue engineering applications. MBGs may be doped with biologically active ions to tailor their biological activity. Boron is being widely studied as a dopant of bioactive glasses. Recently, research has demonstrated the potential of boron-containing bioactive glasses for muscle regeneration. In this study, boron-containing MBGs, 10B-MBG and 18B-MBG nanoparticles, were produced by a microemulsion-assisted sol–gel approach for potential muscle regeneration applications. First, X-ray diffraction (XRD), Fourier transform infrared (FTIR), and energy-dispersive X-ray spectroscopy (EDX) analyses were conducted to study the chemical structure and composition of the nanoparticles. To examine the nanoparticle morphology, scanning electron microscopy (SEM) and transmission electron microscopy (TEM) images were analyzed. Both SEM images and particle size distribution determined by dynamic light scattering (DLS) indicated a decrease of the average particle size after boron doping. TEM images indicated a slit-shaped mesoporous structure of nanoparticles for all compositions. The ζ potential was measured, and a negative surface charge was found for all study groups due to the presence of silanol groups. Cytocompatibility and fluorescence microscopy studies were also carried out. The results indicated that low concentrations (0.1 and 1 mg mL^–1^) of all MBG nanoparticles led to high viability of C2C12 cells. Fluorescence microscopy images indicated that at lower nanoparticle concentrations (0.1 and 1 mg mL^–1^), C2C12 cells appeared to differentiate into myotubes, which was indicated by a spindle-shaped morphology. For 10 mg mL^–1^ concentration of nanoparticles, C2C12 cells had a lower aspect ratio (estimated qualitatively by inspection of the images), which implied a lower degree of differentiation. Boron-doped MBG nanoparticles in reduced concentrations are suitable to induce differentiation of C2C12 cells into myotubes, indicating their potential for applications in muscle tissue repair.

## Introduction

Skeletal muscle is responsible for movement, stabilization of joints, maintenance of posture, and generation of heat.^1,2^ Skeletal muscle has a significant self-healing capacity due to its myogenic stem cells and multipotent muscle satellite cells.^2−4^ However, volumetric muscle loss (VML) occurs if specific muscle loss surpasses 20% of a specific muscle mass.^5−7^ This causes scar tissue formation, which has prompted research activities to investigate methods to establish skeletal muscle regeneration.^8−10^ To treat skeletal muscle, stem cell transplantation^11^ and use of braces^5,12,13^ are current clinical approaches. However, these methods have drawbacks. Stem cell transplantation involves simultaneous application of radiation and toxic drugs, and the use of braces reduces the life quality of patients.^5,11−13^ Tissue engineering is a promising approach to overcome these disadvantages.^10,14^ For this purpose, various scaffolds aiming at muscle tissue regeneration have been fabricated by combining biomaterials, cells, and biologically active molecules.^15−17^

Bioactive glasses have been attracting research interest for biomedical applications since their discovery in the late 1960s.^18−21^ Mesoporous bioactive glass (MBG) nanoparticles in different compositions are promising due to their small particle size and ordered porosity, which makes them attractive for several biomedical applications.^22−25^ Additionally, sol–gel processed MBGs can have higher purity and homogeneity than melt-derived glasses.^26^ Moreover, due to the presence of silanol groups, MBG nanoparticles can be further functionalized.^27^ These particles can be loaded with drugs (antibiotics, growth factors, or enzymes) and biologically active ions to tailor their biological properties.^28−31^ MBGs with certain compositions possess high bioactivity as well as osteogenic and angiogenic properties.^31,32^ Research indicates that boron stimulates bone healing, and it is also promising for soft tissue engineering applications, especially wound healing.^33−37^ Boron’s anti-inflammatory properties are also advantageous for these applications.^33^

Recently, the effect of bioactive glasses on muscle regeneration started to be investigated. Jia et al.^38^ studied the potential of melt-derived 13-93B3 (5.5% Na_2_O, 56% B_2_O_3_, 18.5% CaO, 4.6% MgO, 3.7% P_2_O_5_, 11.1% K_2_O in wt %), 45S5 (24.5% CaO, 24.5% Na_2_O, 45.0% SiO_2_, 6.0% P_2_O_5_ in wt %), and 8A3B (10.8% Al_2_O_3_, 4.9% Na_2_O, 50.7% B_2_O_3_, 16.4% CaO, 4.1% MgO, 3.2% P_2_O_5_, 9.9% K_2_O in wt %) glasses for muscle regeneration. In vitro studies indicated that muscle-related gene expressions (IG-1, growth factor mediating the growth of skeletal muscle tissue; and Cx43, growth factor inducing the expression of MyoD and myogenin) increased more for 13-93B3 and 8A3B than for 45S5 BG.^38−40^ The variation of expression of these genes for different study groups indicated the importance of glass composition on its muscle differentiation capacity in vitro.^38^ Kumar et al.^41^ produced sol–gel-derived borosilicate glasses with a composition of *x*Ag_2_O-(100 – *x*)[45.8CaO-8.4B_2_O_3_-45.8SiO_2_], where *x* was varied as 2, 5, 7.5, and 10 mol %. The study indicated that treatment with 100 μg mL^–1^ borosilicate bioactive glass induced the growth of C2C12 cells into myotubes, which was observed from bright-field photomicrographs of the cells.

In the present study, boron-substituted sol–gel-derived MBG nanoparticles were investigated for their suitability for muscle tissue regeneration. In previous studies, boron was substituted up to 15 mol % in MBGs.^33,42−44^ In this study, a higher mol percent of boron (18 mol %) was also studied to examine its effect on physical and biological properties of the nanoparticles. MBG was considered in the basic composition of 58 mol % SiO_2_ and 42 mol % CaO; moreover, 10 mol % boron-doped MBG (10B-MBG) and 18 mol % boron-doped MBG (18B-MBG) nanoparticles were produced and characterized in terms of their chemical structure and physical properties. Different concentrations of MBG, 10B-MBG, and 18B-MBG nanoparticles were used to treat C2C12 cells, which were investigated in terms of cell viability and differentiation. The effect of MBG nanoparticles on the morphology of C2C12 cells was examined.

## Experimental Section

### Synthesis of Boron-Doped MBG Nanoparticles

**Figure 1:**
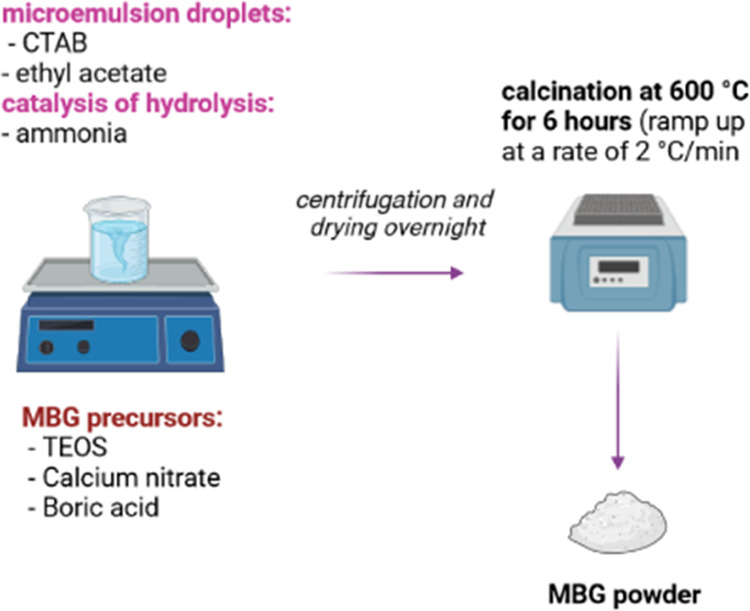
Schematic diagram of MBG nanoparticle synthesis (drawn in Biorender software).

A microemulsion-assisted sol–gel method was used to prepare MBG, 10B-MBG, and 18B-MBG nanoparticles, as shown in Figure 1. Table 1 shows the composition of each study group. To produce 10B-MBG, 4.8 g of hexadecyl trimethyl ammonium bromide (CTAB, BioXtra, 99%, Sigma-Aldrich) was dissolved in 132 mL of deionized water at 37 °C. After the solution became clear, 80 mL of ethyl acetate (99.8%) was added and stirred for 30 min. Then, 3.76 mL of aqueous ammonia (28%) was poured in the solution and stirred for 15 min. This was followed by the addition of 28.8 mL of tetraethyl orthosilicate (TEOS, 99%, Sigma-Aldrich). Next, 24.48 g of calcium nitrate tetrahydrate (99.5%, VWR Chemicals) was added and stirred for 30 min. Finally, 3.2 g of boric acid (≥99.5%, Sigma-Aldrich) was added, and the mixture was further stirred for 4 h. Particles were centrifuged and washed twice with water and once with ethanol (96% VWR Chemicals). Then, they were dried at 60 °C overnight and calcined at 600 °C for 6 h at a heating rate of 2 °C min^–1^.^33,45^

**Table 1:** Nominal mol % of the Components of MBG Nanoparticles

study group	SiO_2_	CaO	B_2_O_3_
MBG	58	42	0
10B-MBG	50	40	10
18B-MBG	45	37	18

### Particle Characterization

X-ray diffraction (XRD) analyses were performed using the X-ray diffractometer MiniFlex 600 (Rigaku) in the 2θ range of 20–80° with Cu Kα radiation. A step size of 0.02° and a dwell time of 1s step^–1^ were used.

Fourier transform infrared spectroscopy (FTIR, IRAffinity-1s spectrometer, SHIMADZU, Japan) was carried out in transmission mode to investigate the chemical structure of the samples. FTIR spectra were obtained at the wavenumber range of 400–2000 cm^–1^.

The MBG nanoparticle morphology was examined by scanning electron microscopy (SEM) (Zeiss Auriga 4750) under an accelerating voltage of 1 kV. Qualitative compositional analysis was carried out by energy-dispersive X-ray spectroscopy (EDX). Samples were sieved and dispersed onto a carbon tape on an aluminum stub without coating. Moreover, transmission electron microscopy (TEM) studies were conducted using an FEI Talos F200S microscope (Thermo Fisher Scientific, Eindhoven, The Netherlands). Samples were dropped on a Holey carbon film on a copper grid before TEM images were acquired.

Dynamic light scattering (DLS) and ζ potential measurements were carried out using a Zetasizer NanoZS (Malvern Instruments, U.K.). Samples were diluted to 0.1% in deionized water before taking measurements.

pH measurements were carried out after addition of 0.05 g of MBG nanoparticles in 5 mL of Dulbecco’s modified Eagle’s medium (DMEM) solution after 0.5, 1, 18, 24, and 48 h.

### In Vitro Cytocompatibility Assay

#### Cell Culture and Maintenance

C2C12 cells (PromoCell GmbH, Heidelberg, Germany) (passage 12–14) were cultured in cell culture flasks (Sarstedt, Nümbrecht, Germany) using a cell culture medium containing Dulbecco’s modified Eagle’s medium (DMEM) supplemented with 1 g L^–1^d-glucose, 0.58 g L^–1^l-glutamine, 110 mg L^–1^ sodium pyruvate, 15 mg L^–1^ phenol red (Thermo Fisher Scientific), 10% fetal bovine serum (FBS) (Corning), and 1% penicillin-streptomycin (Thermo Fisher Scientific). Then, the cells were cultured in an incubator at a humidified atmosphere of 5% CO_2_, 95% humidity, and 37 °C. After reaching confluency, the cells were seeded in 24-well plates at the density of 1 × 10^5^ cells mL^–1^ and incubated under a CO_2_ atmosphere at 37 °C for 1 day.

#### WST Assay

MBG, 10B-MBG, and 18B-MBG nanoparticles were sterilized at 160 °C for 2 h in a furnace (Naberthm, Germany). In 10 mL of the cell culture medium, 100 mg of sample (10 mg mL^–1^) was added and incubated at 37 °C for 24 h. This sample was further diluted to form 1 and 0.1 mg mL^–1^ solutions for each sample group. Each solution was filtered with a 0.2 μm polypropylene sterile syringe filter (Corning) to prepare extracts. The cells that were grown in 24-well plates for 1 day were treated with extracts (*n* = 4) for 2 days. The control group was also prepared, which had cells seeded but had no extract treatment. After this, extracts were removed and 400 μL of the cell culture medium with 2 vol % WST-8 reagent (CCK-8 kit, Sigma-Aldrich) was kept on the cells for 3 h at 37 °C to measure the cell mitochondrial activity.^46^ Depending on percentage cell viability, WST8 (2-(2-methoxy-4-nitrophenyl)-3-(4-nitrophenyl)-5-(2,4-disulfophenyl)-2*H*-tetrazolium) was bioreduced by cellular dehydrogenases to an orange formazan product. From each 24-well plate, 100 μL of the medium was transferred to three wells of 96-well plates, and the absorbance at 450 nm was measured using a well plate reader (type Phomo, Anthos Mikrosysteme GmbH, Krefeld, Germany). Relative % cell viability was determined according to the equation
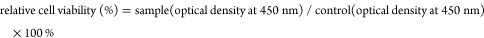


#### Fluorescence Microscopy Imaging

To study the morphology of C2C12 cells after 2 days of treatment with the extracts, cells were stained for F-actin. The cells were fixed using 4% (w/v) paraformaldehyde in phosphate-buffered saline (PBS) for 15 min and permeabilized with a Triton-X 100 containing a permeabilization buffer for 5 min. Then, cells were washed with PBS 3 times and a phalloidin solution (R415, molecular probes, Thermo Fisher Scientific, Germany) was added in the dark for 1 h. Samples were then washed and counterstained with DAPI (4′,6-diamidino-2-phenylindole, dihydrochloride, Thermo Fisher Scientific, Germany) for 5 min. After washing with PBS, fluorescent images were taken with a fluorescence microscope (Axio Observer, Carl Zeiss).^47^ In ImageJ software, the widths of the filaments were measured on approximately 35 filaments.

### Statistics

Quantitative data are reported as mean value ± standard error from at least three independent experiments. Statistical differences between groups were analyzed using the two-way analysis of variance (ANOVA) statistical test, with Tukey’s pairwise post hoc test. Statistical significance is represented as ^#^*p* < 0.05 (in comparison to the control group).

## Results and Discussion

Figure 1 shows a schematic diagram of the preparation of MBG, 10B-MBG, and 18B-MBG nanoparticles by microemulsion-assisted sol–gel processing. CTAB is first added in water, which is followed by the addition of ethyl acetate. Hydrophobic ethyl acetate self-assembles CTAB micelles, forming microemulsion droplets, which act as templates for the formation of the mesoporous structure.^20,48^ Then, aqueous ammonia is added in the solution, which later catalyzes the hydrolysis and condensation of TEOS to form the silica network on the microemulsion droplets.^20,26^ The addition of MBG precursors (TEOS, calcium nitrate, and boric acid) follows, and then, samples are centrifuged, washed, and dried. The final step is the calcination process, which allows elimination of organic impurities.^20^Table 1 shows the composition of each sample group.

**Figure 2:**
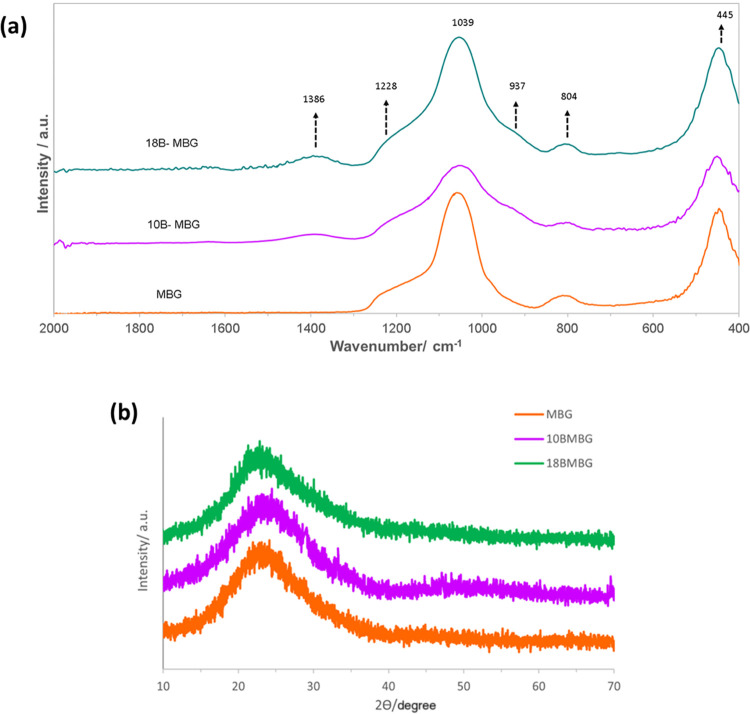
(a) FTIR spectra and (b) XRD patterns of MBG, 10B-MBG, and 18B-MBG nanoparticles (the relevant bands in the FTIR spectra are discussed in the text).

Figure 2a shows the FTIR spectra of MBG, 10B-MBG, and 18B-MBG particles. The band found at 1228 cm^–1^ was attributed to the [SiO_4_] tetrahedral, and the shoulder at 1039 cm^–1^ was due to the Si–O–Si asymmetric stretching mode. The bands at 804 and 445 cm^–1^ are attributed to Si–O–Si symmetric stretching and bending vibrations, respectively. All of these bands confirmed the silicate network formation. After boron substitution, additional bands were formed at 1386 and 937 cm^–1^, which occurred due to B–O–B stretching vibrations of [BO_3_] and stretching vibrations of B–O from BO_4_ units, respectively.^33^ In Figure 2b, XRD results show the broad halo in the results of all samples, which revealed that the samples were amorphous. The results thus prove that boron substitution was successfully achieved in the silicate network.

**Figure 3:**
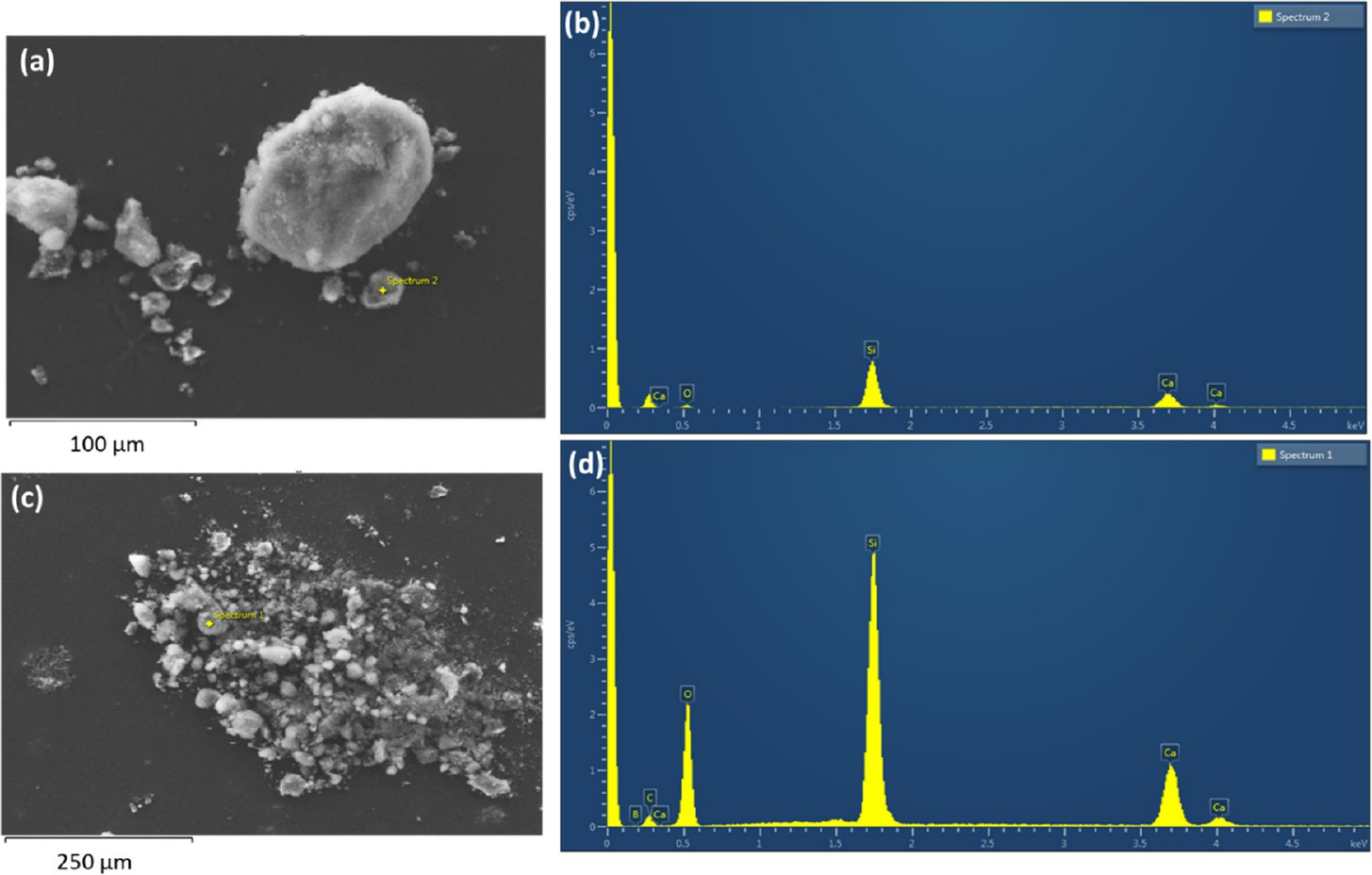
SEM image (a) and EDX analysis (b) of MBG nanoparticle agglomerates and SEM image (c) of 18B-MBG nanoparticle agglomerates with their EDX compositional analysis (d).

**Figure 4:**
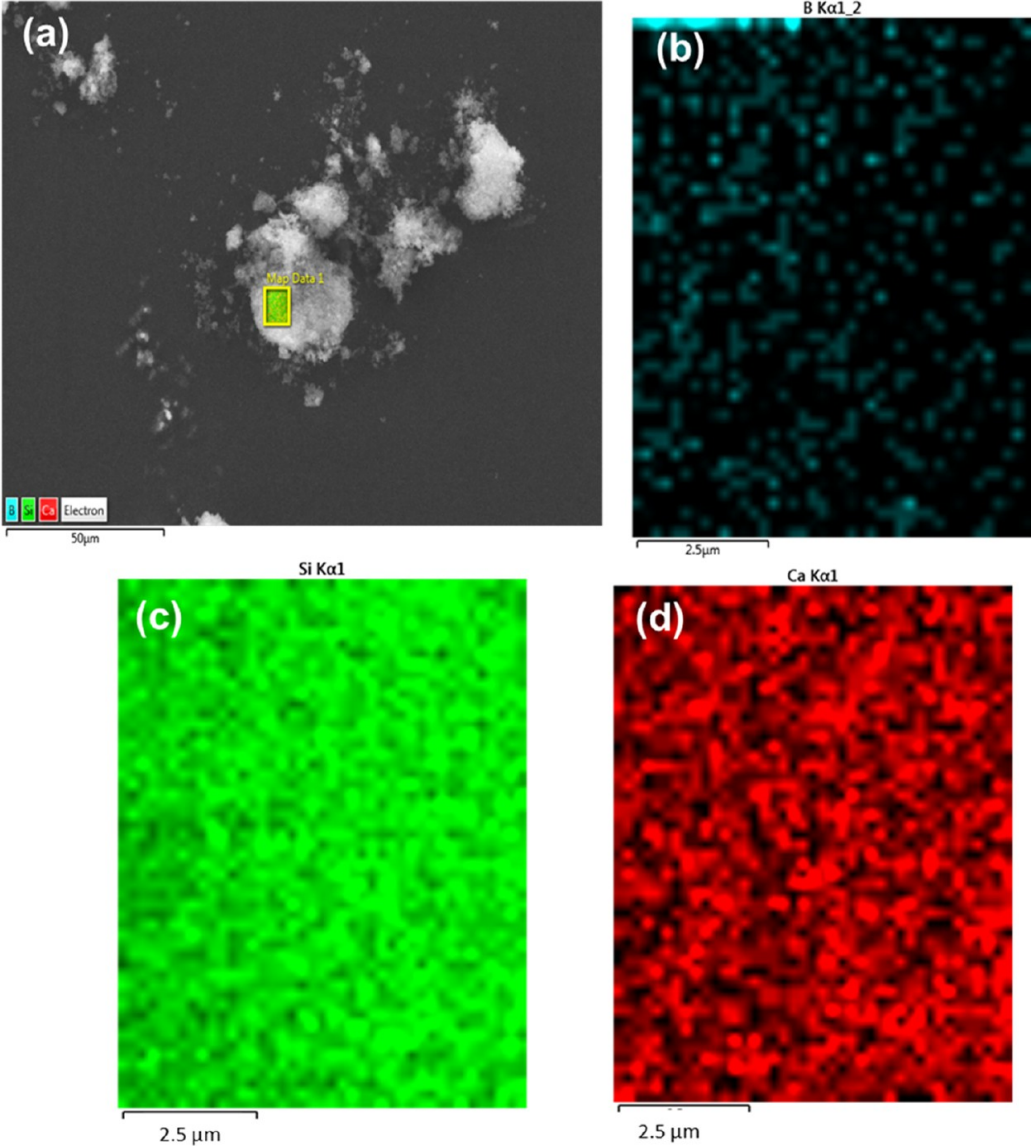
EDX analysis showing the distribution of (a) all ions in the selected region on 18B-MBG nanoparticles, and (b) boron, (c) silicon, and (d) calcium distribution.

Figure 3a demonstrates the presence of only Si and Ca in MBG nanoparticles. In Figure 3b, the EDX spectrum of 18B-MBG indicates the presence of Si, Ca, and B, which also further confirms the substitution of boron in the MBG structure. Figure 4a shows a low-magnification image of MBG nanoparticle agglomerates. Additionally, Figure 4b–d shows an even distribution of Si, Ca, and B (measurement is from the region indicated in Figure 4a), respectively, in 18B-MBG nanoparticles.

Table 2 shows the atomic percentages of boron, silica, calcium, and oxygen ions from EDX data. This data can be used to indicate that boron was successfully incorporated into the MBG structure.

**Table 2:** EDX Data Showing Atomic Percentages of Boron, Silica, and Calcium Ions of the Prepared MBG Particles

spectrum label	MBG	10-MBG	18-MBG
B (%)	0	10.55	16.72
O (%)	59.83	52.61	50.03
Si (%)	27.24	24.21	22.55
Ca (%)	12.93	12.63	10.70
total	100.00	100.00	100.00

**Figure 5:**
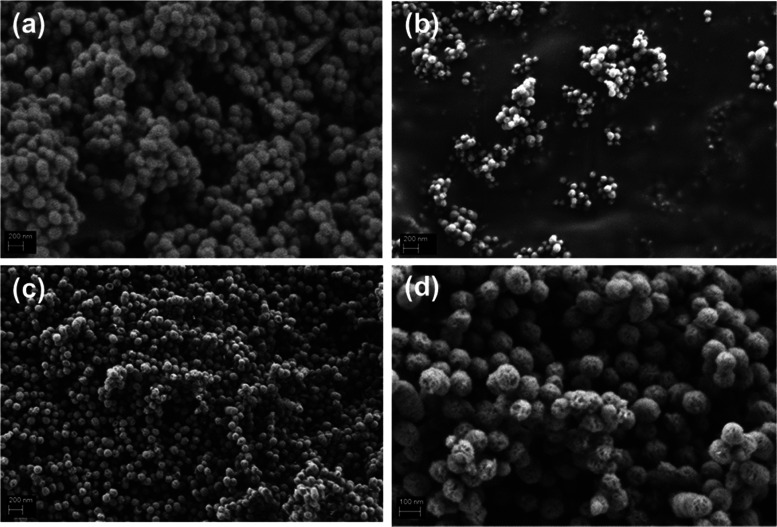
SEM images of (a) MBG, (b) 10B-MBG, (c) 18B-MBG nanoparticles, and (d) 18B-MBG nanoparticles at higher magnification.

Figure 5 shows SEM images of the nanoparticles. The images reveal that after incorporation of boron ions, a decrease of particle size occurred. Despite the change of size of the particles, the spherical morphology of MBG nanoparticles was sustained. Also, the internal porosity of 18B-MBG nanoparticles can be observed at high magnification (Figure 5d).

**Figure 6:**
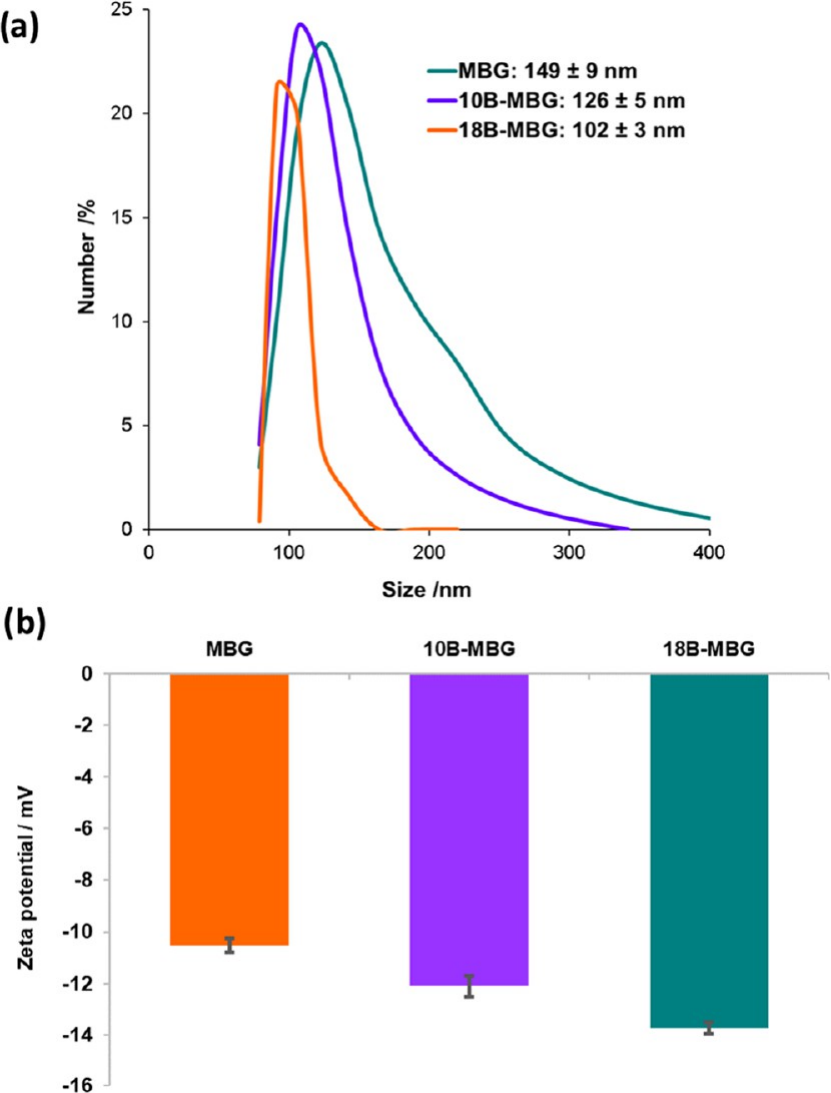
(a) Particle size distribution obtained by DLS measurements, and (b) ζ potentials of MBG, 10B-MBG, and 18B-MBG particles.

Figure 6a shows the size distribution of the nanoparticles, which was measured by DLS. The results demonstrate the narrow particle size distribution. With the increase of boron concentration, the particle size distribution became narrower. Also, the particle size was reduced after doping with boron ions. The reduction of particle size after boron doping was also evident from the SEM images in Figure 5. Yang et al.^49^ also observed a reduction of particle size with the increase of boron content, and this was attributed to the change in the nature of bonding in the silica network. This effect could be due to changes in the rate of hydrolysis and condensation reactions with the addition of a boric acid precursor.^26,50^Figure 6b shows the ζ potential values measured on the nanoparticles. The negative surface charge is ascribed to silanol groups on the particle surfaces. The value further decreased with boron substitution, and this could be due to the reduction of particle size, which led to a higher surface area and a higher concentration of silanol groups.

**Figure 7:**
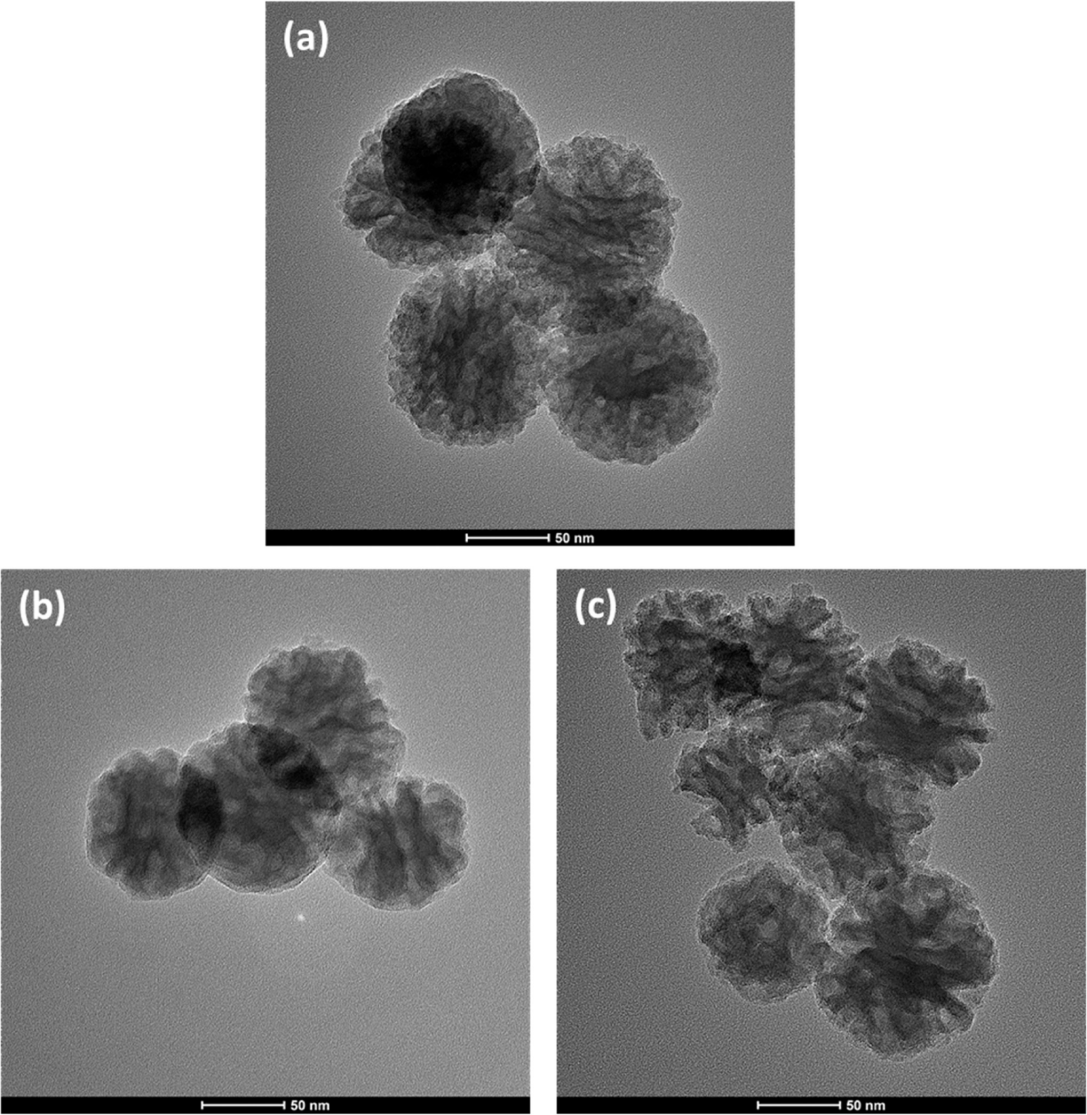
TEM images of (a) MBG, (b) 10B-MBG, and (c) 15B-MBG nanoparticles.

Figure 7 shows the TEM images of MBG, 10B-MBG, and 18M-MBG nanoparticles. These images demonstrate the slit-shaped mesoporous structure of all particles with different compositions. The images demonstrate that the incorporation of boron into the structure did not alter the shape of the nanoparticles. In a previous study by Zheng et al.,^33^ boron-doped MBG was prepared with the same method, and the authors carried out Brunauer–Emmett–Teller (BET) studies. A type IV nitrogen isotherm with an H3 hysteresis loop indicated a mesoporous structure with slit-shaped pores. Furthermore, the pore size was observed to increase with the increase of boron concentration from 10 to 15%. From the TEM images of this study, it is also evident that the pore size increased with the addition of boron ions in the MBG structure.

**Figure 8:**
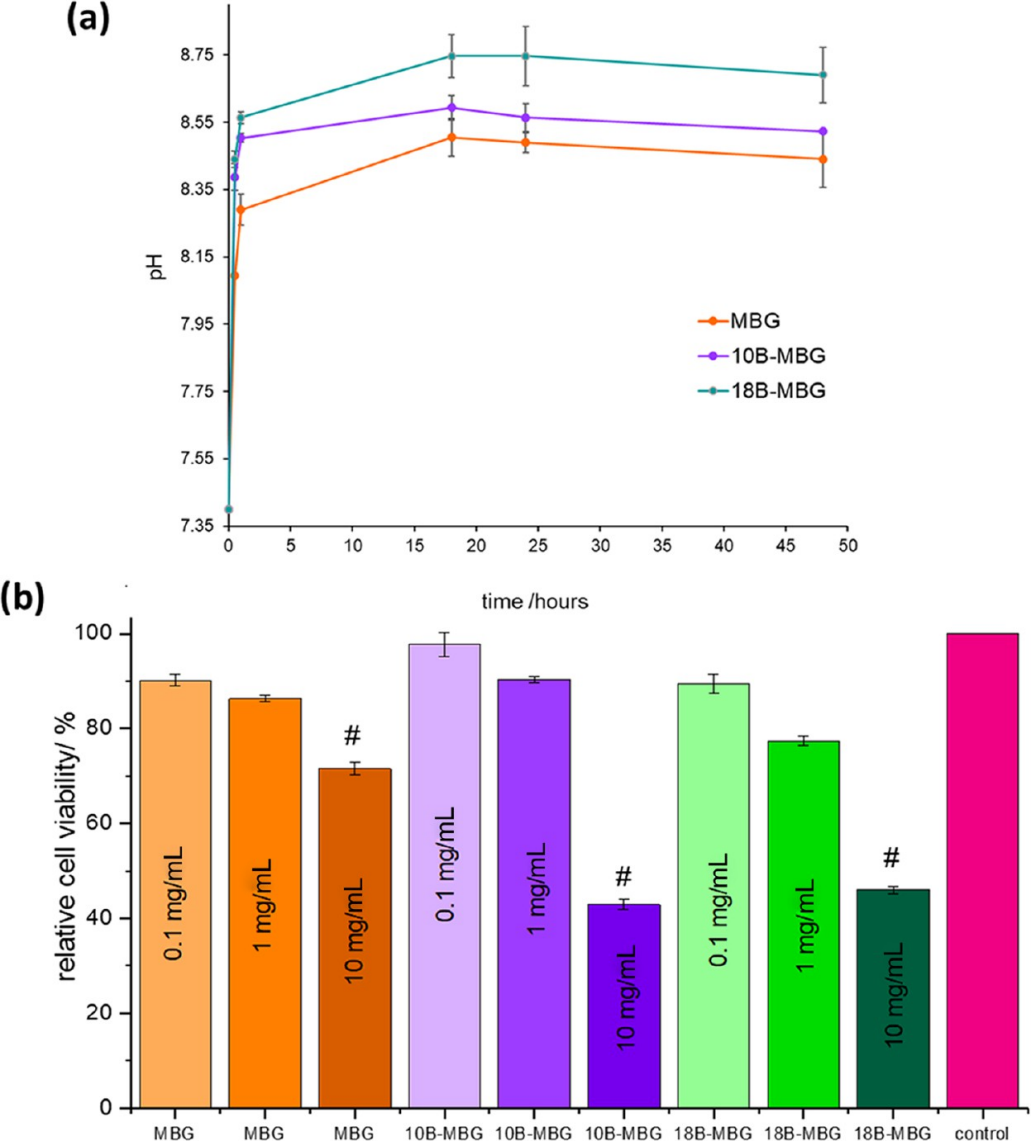
(a) pH measurement results for MBG, 10B-MBG, and 18B-MBG nanoparticles in DMEM. (b) Relative viability of C2C12 cells cultured with extracts of MBG, 10B-MBG, and 18B-MBG at concentrations of 0.1, 1, and 10 mg mL^–1^ after 2 days of culture (^#^*p* < 0.05 in comparison to the control group).

In Figure 8a, it is observed that there is an abrupt increase of pH in the initial stage of immersion in DMEM. This occurs due to calcium ion exchange with hydrogen ions immediately after immersion of the particles in DMEM.^51−54^ There is an increase of pH with the increase of mol % of boron in MBG particles. The studies indicate that the pH of the solution increases more rapidly when the boron content of the glass increases. This may be due to the lower network connectivity after boron addition leading to the faster ion dissolution.^55,56^

Figure 8b shows the relative percentage viability of C2C12 cells after an indirect cell culture study. According to the international standard ISO 10993-5: 2009, a percentage cell viability below 70% indicates cytotoxicity. In the present experiment, after the treatment with the highest concentration (10 mg mL^–1^) of nanoparticle extracts, the percentage cell viability was significantly reduced for all study groups. The percentage cell viability was further reduced for both boron-containing groups (10B-MBG and 18B-MBG) compared with the pure MBG study group. This is probably due to induction of cytotoxicity due to the higher concentration of [BO_3_]^3–^.^57^ Also, as discussed earlier, boron substitution increased the pH, which may lead to the increase of cytotoxicity. For lower concentrations (0.1 and 1 mg mL^–1^ MBG), the percentage cell viability was found to have no significant difference compared with the control group, and all study groups were found to be cytocompatible.

Literature reports have shown that silica nanoparticles induce myoblast fusion in C2C12 cells.^9,58^ The aspect ratio of cells, which is the ratio of the long axis to the short axis of the cells, is an important indicator of morphological changes.^59^ An increase of the aspect ratio of C2C12 cells occurs with their differentiation to myotubes.^60,61^ Bruyère et al.^62^ considered the aspect ratio to study the fusion of myoblast into myotubes. This process occurs “by elongation of myoblasts into a bipolar shape”. In this study, the aspect ratio was also examined to evaluate the degree of differentiation of C2C12 cells into myotubes.

**Figure 9:**
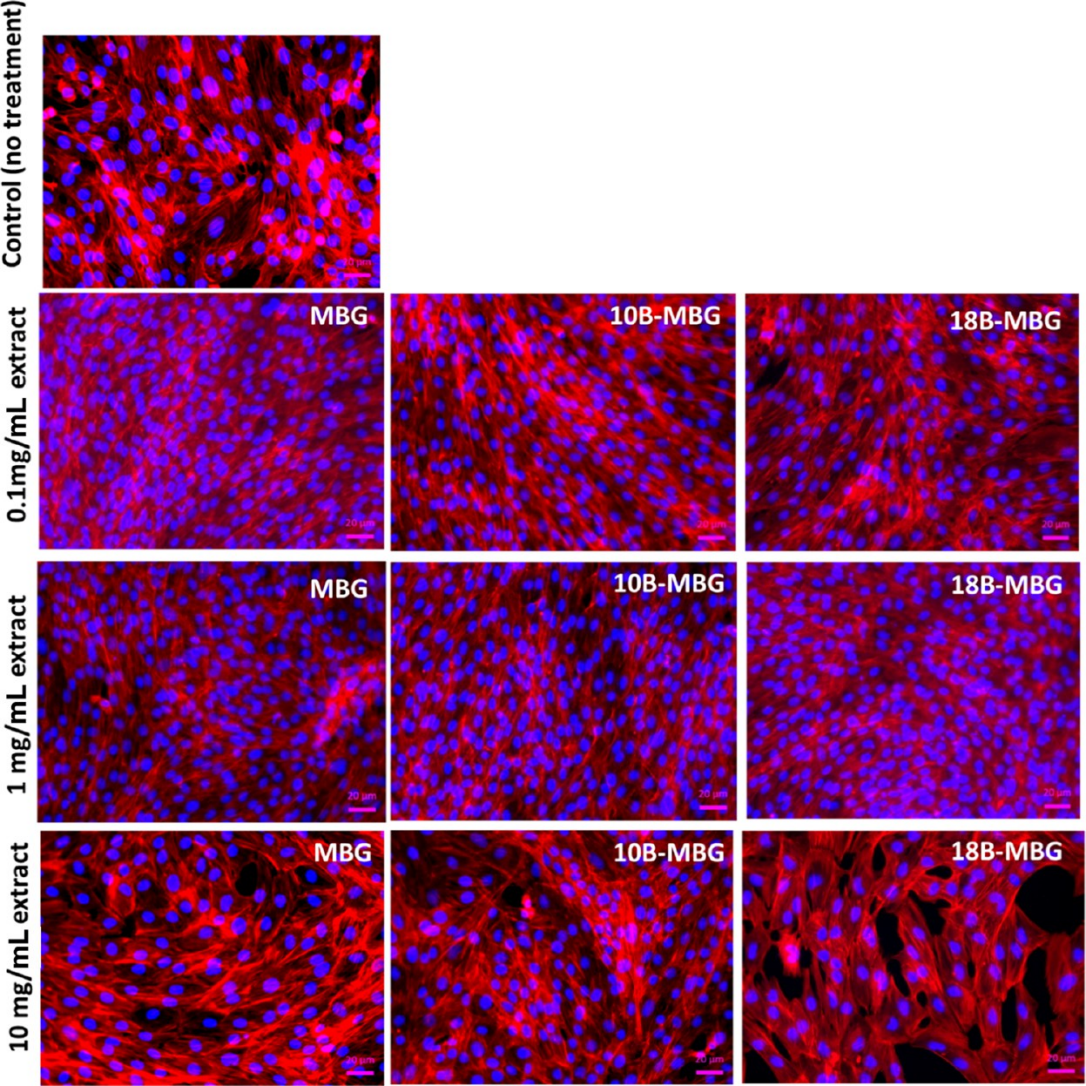
Fluorescence microscopy images of C2C12 cells after 48 h of indirect treatment with MBG dissolution products after staining F-actin with phalloidin red and nuclei with DAPI (control group: no extract treatment) (scale bar = 100 μm).

In 24-well plates, within 3 days, all study groups started to differentiate; however, there were distinct differences between study groups. In Figure 9, fluorescence microscopy images illustrate qualitatively that a spindle-like morphology appears for C2C12 cells treated with 0.1 and 1 mg mL^–1^ MBG nanoparticle extracts. For 10 mg mL^–1^ treatment with extracts, the actin filaments have a relatively larger width and they are less elongated.

**Figure 10:**
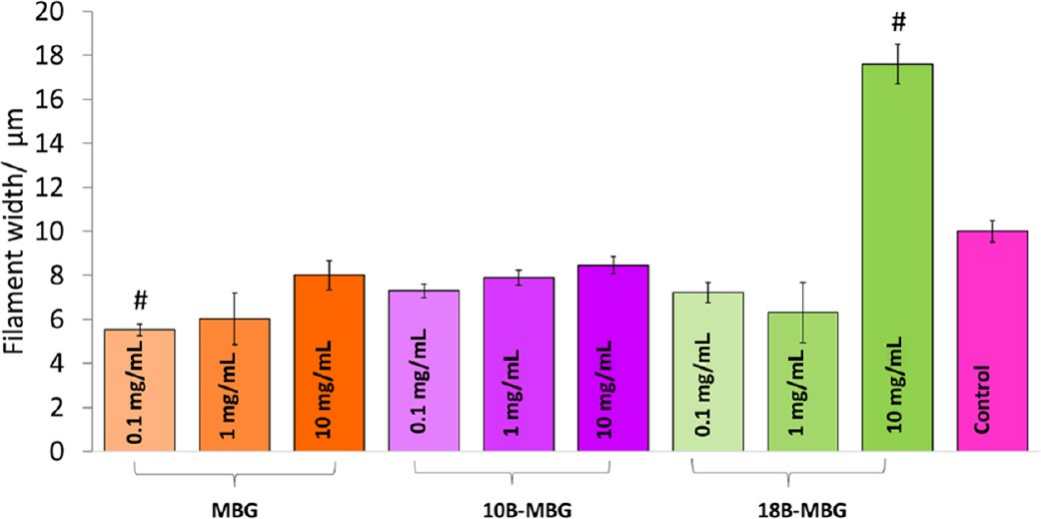
Width of actin filaments of C2C12 cells after exposure to dissolution products of MBG, 10B-MBG, and 18B-MBG nanoparticles with different concentrations for 48 h (measurements were made using ImageJ software) (#*p* < 0.05 in comparison to the control group) (*n* = 35).

Along with measurement of the width of actin filaments using ImageJ software from fluorescence microscopy images, Figures 9 and 10 indicate that treatment with 1 mg mL^–1^ MBG and 10B-MBG nanoparticle extracts significantly increased the aspect ratio of the cells compared to the control group. This increase of aspect ratio was more pronounced after the 0.1 mg mL^–1^ pure MBG nanoparticle extract treatment. After the treatment with 10 mg mL^–1^ 18B-MBG nanoparticle extracts, the aspect ratios were significantly reduced. This result indicates that C2C12 cells exposed to a high concentration of ions did not undergo differentiation as much as cells treated with 0.1 and 1 mg mL^–1^ MBG, 10B-MBG, and 18B-MBG nanoparticle extracts.

For the 10 mg mL^–1^ 18B-MBG extract, treated cells exhibited a 77% increase of the actin filament width compared to the control group. On the other hand, cells treated with the 0.1 mg mL^–1^ 10B-MBG extract showed a 45% reduction of the actin filament width compared to the control group. This result shows a distinct effect of the concentration of released ions on the cell morphology. The increase of filament width and the decrease of aspect ratio are probably due to cytotoxic effects of boron ions at high concentrations, which reduced the percentage viability of the cells and ultimately inhibited the differentiation of C2C12 cells, which is initiated at high cell densities.^63,64^

In this study, the produced (MBG) nanoparticles had a relatively high mol % of boron substitution (up to 18 mol %). In the future, MBG particles with a lower boron substitution must be studied to evaluate their effect on C2C12 cell viability and differentiation. This may lead to further optimization of processing conditions of boron-doped MBG particles for potential muscle regeneration applications. MyCH staining is an important indicator of differentiation of C2C12 cells to myotubes. This technique requires to be practiced to further evaluate the effect of differentiation of boron-doped MBG on C2C12 differentiation. Moreover, gene expressions of IG-1 and Cx43 must be studied to further evaluate the effect of boron doping of MBG on C2C12 cell behavior. Finally, a future work on bulk RNA sequencing would give more detailed information about the effect of boron-doped MBG on muscle-related genes. Having confirmed an effect of boron ion release in this study, it can be anticipated that boron-doped MBG particles will show promise for future muscle regeneration applications.

## Conclusions

In this study, MBG, 10B-MBG, and 18B-MBG nanoparticles were produced by a microemulsion-assisted sol–gel approach. FTIR, XRD, and EDX analyses demonstrated the successful incorporation of boron ions in the MBG structure. SEM and DLS analyses demonstrated reduction of particle size with boron substitution. TEM analyses indicated that after incorporation of boron, the slit-shaped mesoporous structure of MBG nanoparticles was sustained. The ζ potential was increased with the addition of boron, which was probably due to the reduction of particle size and the greater surface area. Concentrations of 0.1 and 1 mg mL^–1^ of all nanoparticle extracts were found to be cytocompatible. Furthermore, MBG, 10B-MBG, and 18B-MBG nanoparticle extracts with 0.1 and 1 mg mL^–1^ concentrations induced differentiation of C2C12 cells into myotubes. Future research is required for investigating MBG nanoparticles with a lower boron substitution to evaluate their effect on muscle cell differentiation. The potential of boron-doped MBG for muscle tissue repair applications remains an interesting novel area for further investigations.
